# Geomagnetic disturbances and grid vulnerability: Correlating storm intensity with power system failures

**DOI:** 10.1371/journal.pone.0327716

**Published:** 2025-07-28

**Authors:** Mauro González Figueroa, Daniel David Herrera Acevedo, David Sierra Porta

**Affiliations:** 1 Escuela de Ingeniería, Arquitectura y Dise no, Universidad Tecnológica de Bolívar, Cartagena de Indias, Bolívar, Colombia; 2 Escuela de Transformación Digital, Universidad Tecnológica de Bolívar, Cartagena de Indias, Bolívar, Colombia; Instituto Nacional de Pesquisas Espaciais, BRAZIL

## Abstract

Geomagnetic storms represent a critical yet sometimes overlooked factor affecting the reliability of modern power systems. This study examines the relationship between geomagnetic storm activity—characterized by the Dst index and categorized into weak, moderate, strong, severe, and extreme intensities—and reported power outages of unknown or unusual origin in the United States from 2006 to 2023. Outage data come from the DOE OE-417 Annual Summaries, while heliospheric and solar wind parameters (including proton density, plasma speed, and the interplanetary magnetic field) were obtained from NASA’s OMNIWeb database. Results indicate that years with a higher total count of geomagnetic storms, especially those featuring multiple strong or severe events, exhibit elevated incidences of unexplained power interruptions. Correlation analyses further reveal that increasingly negative Dst values, enhanced solar wind velocity, and higher alpha/proton ratios align with greater numbers of outages attributed to unknown causes, underscoring the pivotal role of solar wind–magnetosphere coupling. A simple regression model confirms that storm intensity and average magnetic field strength are statistically significant predictors of unexplained outages, more so than broad indicators such as sunspot number alone. These findings highlight the importance of monitoring high-intensity geomagnetic storms and associated heliospheric variables to mitigate potential risks. Greater attention to space weather impacts and improved reporting of outage causes could bolster grid resilience, helping operators anticipate and manage disruptions linked to geomagnetic disturbances.

## Introduction

The modern world depends on a continuous and reliable supply of electric power for communication systems, transportation networks, industrial production, and household activities. Contemporary power systems, which encompass both generation and transmission infrastructures, are highly optimized networks that operate under precise conditions. Despite their robustness, these systems remain vulnerable to a variety of external disruptions [1–3], including severe weather events [4,5], physical attacks [6], equipment failures [7,8], and geomagnetic storms, for which empirical data on grid impacts are relatively scarce compared to terrestrial weather phenomena [9,10].

Geomagnetic storms are driven primarily by large-scale solar wind structures—namely coronal mass ejections (CMEs) and high-speed streams from coronal holes—that carry prolonged southward interplanetary magnetic field (IMF *B*_*z*_<0), rather than by impulsive solar flares alone [11–13]. When the IMF turns and remains southward, dayside reconnection opens the magnetosphere–ionosphere system, injecting energy and momentum into the ring current and driving the characteristic depressions of the Dst index. Rapid temporal variations in Earth’s magnetic field (∂B/∂t) then induce geoelectric fields at ground level via Faraday’s law, which drive quasi-DC currents through any sufficiently long conductive path—transmission lines, pipelines, and transformer neutrals—in a process known as Geomagnetically Induced Currents (GICs) [14,15]. These GICs bias three-phase transformer windings with a DC component that produces half-cycle core saturation, generates harmonics, increases reactive power consumption, and causes localized heating of windings and structural components; they can also trip protective relays or cause permanent insulation damage [16,17]. As modern grids have grown in scale and interconnectivity, the extended conductive spans and ground-return paths lengthen GIC flow channels, amplifying current amplitudes and elevating the risk that intense storms will precipitate wide-area electric service disruptions.

The impact of geomagnetic storms on power systems is multifaceted. High-voltage transformers, especially those at higher latitudes, are particularly susceptible to GICs, which can cause core saturation, overheating, and insulation damage, occasionally leading to catastrophic transformer failures [17–20]. Such failures may trigger cascading outages across wide areas. In addition, GICs can interfere with protective relays, communication links, and control systems, thereby intensifying the overall risk.

Historical events such as the 1989 Quebec blackout illustrate the severe consequences of geomagnetic storms. During that event, an intense storm initiated failures in the Hydro-Québec power grid [16,21,22], leaving millions of customers without electricity for several hours. Storms of this magnitude are exceptionally rare: Hayakawa *et al*. (2019) show that only a handful of Carrington-class and sub-Carrington events have occurred since 1859 based on reconstructions of large sunspot groups and auroral records [23]. Nevertheless, more moderate geomagnetic disturbances (Dst≈–50 to –100 nT) occur several times per solar cycle and can still challenge modern grids, particularly as reliance on digital and highly interconnected infrastructures grows [24,25].

The geomagnetic storm of 13 March 1989 produced one of the most disruptive space-weather events in modern history. A fast-moving CME drove an abrupt southward turn of the IMF, inducing quasi-dc currents in the Hydro-Québec 735 kV and 315 kV networks that triggered protective relays within two minutes and plunged six million customers into darkness [22]. In Canada alone, approximately 19400 MW of generation was lost, together with 1326 MW of exports to the U.S.; additional available capacity could not be accessed due to widespread distribution-system failures [22]. The consequent reactive-power shortage led to a near-total voltage collapse of the Hydro-Québec grid. Equipment damage costs were initially estimated at CAD 6.5 million, with the net financial impact to Hydro-Québec (including business-loss claims) reaching CAD 13.2 million [26]. The storm’s geoelectric intensity was exceptional—Dst dipped to approximately –589 nT, and dB/dt at North American observatories peaked between 300 and 600 nT min^−1^ [27]—underscoring the event’s extreme magnitude and its ability to overwhelm both hardware and protection schemes.

Beyond Québec, the storm’s effects spanned the continent and beyond. A 500 kV transformer at the Salem Nuclear Generating Station in New Jersey failed under GIC stress [28], and operational satellites experienced increased atmospheric drag, orbit perturbations, and disruptions to telecommunications and navigation systems [14,22]. Auroral displays were recorded at unusually low latitudes—down to the Tropic of Capricorn in Australia and as far south as Mexico and the Cayman Islands—highlighting the global reach of this single solar wind driver [23]. Together, these impacts reveal how extreme geomagnetic storms can cascade through generation, transmission, distribution, and even space-based infrastructure, validating the urgent need for real-time GIC monitoring and robust mitigation strategies.

The May 2024 geomagnetic storm, classified as G5—the highest on the geomagnetic storm scale—had the potential to cause severe damage to power infrastructure. While no major power grid failures were reported, storms of this magnitude can induce transformer overloads, disrupt control systems, and degrade insulation, leading to extended blackouts and costly repairs [29–32].

On 10 May 2024, a severe geomagnetic storm (Dst≈–420 nT) coincided with Mother’s Day in Mexico and persisted for over 2 hours. This was the most intense event since 2003, driven by a fast CME from AR 3664 that merged with a high-speed solar wind stream. According to Caraballo *et al*. (2025) [33], this storm produced the largest ever recorded GIC amplitudes in Latin American power networks, with peak neutral currents exceeding 30 A at the Mazatlán (Sinaloa), Laguna Verde (Veracruz) and Riviera Maya (Quintana Roo). The Mexico Space Weather Service (SCIESMEX) and the National Space Weather Laboratory had anticipated such extreme conditions and deployed magnetometers, magnetotelluric arrays, and GIC monitors. These measurements captured detailed geoelectric variations and the consequent transformer biases, yielding unprecedented insights into low-latitude GIC dynamics [33,34].

The GIC measurements captured at Mexico’s 400 kV substations during the Mother’s Day storm reveal how even sub-auroral networks can experience significant quasi-DC currents. Although the observed peak currents fell short of levels known to cause immediate hardware failures, the potential cumulative wear from recurrent GIC events remains an open question. Crucially, the strong match between field observations and predictions from the ground-conductivity–tuned induction model in last reference, not only bolsters confidence in its accuracy but also enhances our understanding of the Mexican grid’s dynamic response to severe geomagnetic forcing.

Building on previous efforts to model and document space-weather impacts, this study combines the U.S. DOE’s OE-417 Annual Summaries of emergency incidents with the Dst index to explore empirical correlations between geomagnetic storm intensity and grid disturbances. By examining whether days marked by more intense storms correspond to higher frequencies or severities of reported outages and equipment malfunctions across generation and transmission facilities, we aim to contribute an additional data-driven perspective to ongoing research on grid resilience under space-weather forcing [35–37].

## Data, methods and data mining

### Electric service interruption data (2006–2023)

The primary dataset on electric service interruptions encompasses reports from 2006 up to and including 2023 year. The dataset used corresponds to data tables of reported electrical emergency incidents and disturbances. This dataset collects the annual summary, day-by-day when an interruption occurs, of reported electrical incidents. The data are part of the management of the Office of Cybersecurity, Energy Security and Emergency Response, using the DOE 417 format used by power plant operators to report incidents and unusual normal operating events (https://www.oe.netl.doe.gov/OE417_annual_summary.aspx). Each record provides detailed information on the nature and duration of power outages, capturing both geographical and operational aspects of the events. Specifically, the dataset includes columns for the event month, date and time the event began, date and time of restoration, area affected, North American Electric Reliability Corporation (NERC) region, alert criteria, event type, demand loss in megawatts, and number of customers affected. The resolution is primarily event-based rather than uniformly sampled over time, so outages are recorded when they occur, resulting in daily or finer time granularity.

For subsequent analyses each OE-417 incident was assigned to one of four broad categories—weather, equipment, human, or other/unknown—so that events plausibly linked to space weather (contained in the last category) could be evaluated separately from routine meteorological or operational disturbances.

### Geomagnetic storm data

Heliospheric parameters were taken from the 1-hour OMNI2 data set available via OMNIWeb (https://omniweb.gsfc.nasa.gov/ow.html and straightforward from request data center: https://omniweb.gsfc.nasa.gov/form/dx1.html); this archive provides hourly averages of solar-wind speed, proton density, dynamic pressure, and IMF components in GSM coordinates. The geomagnetic disturbance level was quantified with the hourly Dst index contained in the same OMNI2 files under the field “Dst (Kyoto)”; this column is a direct pass-through of the official Dst series maintained by the World Data Center for Geomagnetism, Kyoto (https://wdc.kugi.kyoto-u.ac.jp/dstdir/). Using coincident 1-hour resolution for all variables ensured proper temporal alignment before aggregation to daily and annual scales.

Geomagnetic storms typically progress through three well-defined stages [38–40]. The first is the storm-sudden-commencement (SSC), seen as an abrupt positive jump in both the hourly Dst index and its 1-minute high-resolution analogue SYM-H, each derived from the same quartet of low-latitude magnetometers [41,42]. Statistical surveys of several hundred SSCs show amplitudes that most often fall in the 10–60 nT range, with medians near 25 nT and rise times of only 1–3 min [43–46].

During the main phase the ring current intensifies and Dst drops below -50 nT for roughly 2-8 h; severe events such as March 1989 reach minima near -589 nT, and historical reconstructions place the 30 October 1903 and 1 September 1859 (Carrington) storms at about -530 nT and -900 nT, respectively [23,47–50]. The subsequent recovery phase restores Dst toward quiet levels over one to several days as the ring current decays. Throughout this study we adopt -50 nT as the operational storm threshold; applying it to the hourly Kyoto Dst series yields a consistent event catalogue for comparison with annual outage statistics.

Geomagnetic activity is often classified according to thresholds in the Dst index, which reflects the intensity of storm-induced variations in Earth’s magnetic field. A typical scheme may treat values dipping below –50 nT as indicating a geomagnetic storm, with further subdivisions based on how negative the index becomes. For instance, According to a widely used categorization by [51] (see also: [52,53]), geomagnetic storms are grouped into categories based on the minimum Dst values attained during an event. Weak storms register between –30 nT and –50 nT, representing relatively minor disturbances in Earth’s magnetosphere. Moderate storms, defined by Dst values from –50 nT to –100 nT, can produce more noticeable effects on technological systems. Strong storms fall within the range of –100 nT to –200 nT, while severe storms extend from –200 nT to –350 nT, indicating substantial disruptions to geomagnetic conditions. Finally, great storms, with Dst values below –350 nT, represent the most extreme and potentially hazardous geomagnetic events observed.

### Data preparation and integrated analysis

Both datasets are aligned chronologically so that each reported power outage can be considered in relation to the corresponding intervals of geomagnetic activity. Special attention is devoted to matching dates and times of outages with periods of elevated solar wind–magnetosphere coupling indicated by the Dst index. In practice, this requires resampling and time-windowing of the minute-resolution Dst measurements to match the event-based nature of outage reports.

Geomagnetic storms typically evolve over several hours, beginning with an initial drop in the Dst index that signals intensifying solar wind–magnetosphere coupling. Depending on the storm’s intensity, this coupling may produce adverse effects on Earth’s ionosphere and thermosphere within a few hours—often ranging from 1 to 2 hours, yet potentially extending up to 12 hours for particularly strong or prolonged disturbances [29,54,55]. Accordingly, infrastructure impacts such as unexpected power outages may occur not only at the peak of the disturbance but also during the storm’s buildup or recovery phases.

In recognition of these dynamics, we selected a 24-hour window around each storm’s main phase as a sufficient interval to capture both near-immediate disruptions and those that might emerge with a delay. Specifically, for each day in which the Dst index dipped below -50 nT (indicating the onset of a storm), we matched any reported outage event occurring from 24 hours before to 24 hours after that day’s main phase. We also tested narrower (±12-hour) and broader (±72-hour) windows; the overall correlation patterns remained consistent, reinforcing the choice of a 24-hour window as a balanced approach to account for both acute and somewhat delayed storm-related impacts.

Each outage is then linked to the most appropriate cause category, ensuring that the classification remains consistent across multiple sources and reporting standards. This classification process is carried out in two stages. The first stage involves an automated data mining approach supported by a Natural Language Processing (NLP) algorithm, which identifies keywords or phrases that strongly correlate with specific categories. For instance, the appearance of terms such as “cold storm” would be flagged as a weather-related event, while instances of “vandalism” would be mapped to human factors. In the second stage, any outages that remain ambiguous after the automated analysis are examined on a case-by-case basis. Domain experts review the context and supporting information, assigning a definitive category once the nature of the incident is clarified.

This integrated approach allows us to explore how the frequency and severity of geomagnetic storms can coincide with, or potentially exacerbate, the risk of large-scale power interruptions in the United States. By characterizing outage events according to likely cause and categorizing geomagnetic activity by Dst-based metrics, the study aims to provide a clearer understanding of the relationships between solar-driven phenomena and electric grid vulnerabilities.

### Event–study design at daily resolution

While annual and monthly aggregates are well suited for long-term correlations, assessing short-term causality demands a finer temporal grid. We therefore implemented a daily *event-study* framework that explicitly tests whether unexplained outages cluster in the narrow time windows surrounding geomagnetic storms.

**Storm events.** Using the hourly OMNIWeb catalogue (Dst index), we defined **storm onset** as the first hour in which Dst<−50nT after at least three consecutive hours with Dst≥−50nT. Onsets were identified between 1 January 2016 and 31 December 2023; hours belonging to the same disturbance phase and separated by fewer than 24 h were merged to avoid double counting. For robustness we later repeated the analysis with a stricter threshold Dst<−100nT (see section below).

**Construction of the daily panel.** Let 𝒟 be the set of calendar days in the study period. For each day t∈𝒟 we created two binary indicators:

1. storm_window_*t*_ = 1 if *t* lies within ±d days of any storm onset (d=1,2,3,4 tested independently), and 0 otherwise;
2. outage_unknown_*t*_ = 1 if at least one OE–417 report classified as Unknown or Other started on day *t*, and 0 otherwise. Multiple outages on the same day are collapsed into a single event.

The resulting panel comprises |𝒟|=2922 rows and is free of missing entries because both indicators are deterministically defined.

**Statistical test.** For each window size we formed a 2×2 contingency table with rows {*storm*, *quiet*} and columns {*outage*, *no outage*}. Independence was evaluated with Pearson’s $\chi^{2}$ statistic; when any expected cell count fell below 5 the exact Fisher test was substituted. Effect magnitude is reported as a risk ratio RR=P(outage∣storm)/P(outage∣quiet) and its Wald 95 % confidence interval.

**Logistic model.** To verify that the excess risk is not driven by seasonality, we fitted a logistic regression with month dummies:

$$logit[P(\text{outage})]=\beta_{0}+\beta_{1}\;{\text{storm\_window}}_{\pm 2}+\sum_{m=1}^{11}\gamma_{m}\;{\text{Month}}_{m}.$$ (1)

Including year dummies proved unstable owing to the small number of outage days; the month-adjusted odds ratio nevertheless remains close to the unadjusted estimate (see Sect).

All analyses were executed in Python with pandas, scipy and statsmodels; fully reproducible code is available in the repository cited under “Data Availability”.

## Results and discussions

### Annual-scale correlations between geomagnetic activity and unexplained outages

The dataset integrates multiple solar and geomagnetic parameters, as well as annual counts of power interruptions reported under unknown or unusual circumstances. These parameters include the Disturbance Storm Time (Dst) index, yearly sunspot numbers (R), solar radio flux at 10.7 cm (F10.7), average proton temperature and density, mean plasma velocity, the alpha–proton ratio, flow pressure, and the interplanetary magnetic field (IMF). In addition, the dataset tracks the yearly counts of geomagnetic storms categorized as weak, moderate, strong, severe, or extreme according to Dst thresholds, as well as the total number of geomagnetic storms (sum of all categories). Finally, each record includes the corresponding year and the number of electrical outages attributed to unknown causes that do not clearly fit into weather-related, equipment-related, or anthropogenic (e.g., vandalism) events.

**Fig 1:**
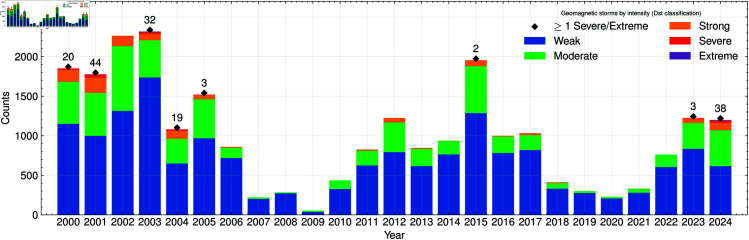
Geomagnetic storm counts by intensity. Stacked bar chart showing the annual counts of geomagnetic storms from 2000 to 2023, categorized by intensity (weak, moderate, strong, severe, and extreme). The total height of each bar represents the sum of all storms in a given year. The figure shows a mark when a Severe or Extreme geomagnetic storm has occurred due to the low and unusual occurrence of these compared to the other classes.

Analysis of this integrated dataset reveals that the total number of geomagnetic storms per year (sum of weak, moderate, strong, severe, and extreme storms) generally follows expected patterns of solar activity, with higher counts often aligning with periods of elevated sunspot numbers (R) and solar radio flux (F10.7) [56,57]. In Fig 1, the stacked bars illustrate the distribution of storms by intensity category for each year from 2000 through 2023, highlighting the variability of geomagnetic activity across solar cycles.

Although weak storms make up the largest portion in most years, several noteworthy peaks stand out in the moderate, strong, and severe categories. In particular, 2015 exhibits the highest overall storm count, with a substantial contribution from moderate storms, as well as a smaller but significant presence of strong and severe events. Years such as 2011 and 2014 also feature an elevated number of moderate storms, while 2013 and 2016 show visibly distinct segments of strong storms (green bars), suggesting heightened geomagnetic activity during these periods. Although severe storms (red bars) are relatively rare, small but noticeable occurrences are visible in 2013, 2015, 2016, and 2023, highlighting pockets of intense geomagnetic disturbance that potentially pose greater risks to power grid infrastructure.

**Fig 2:**
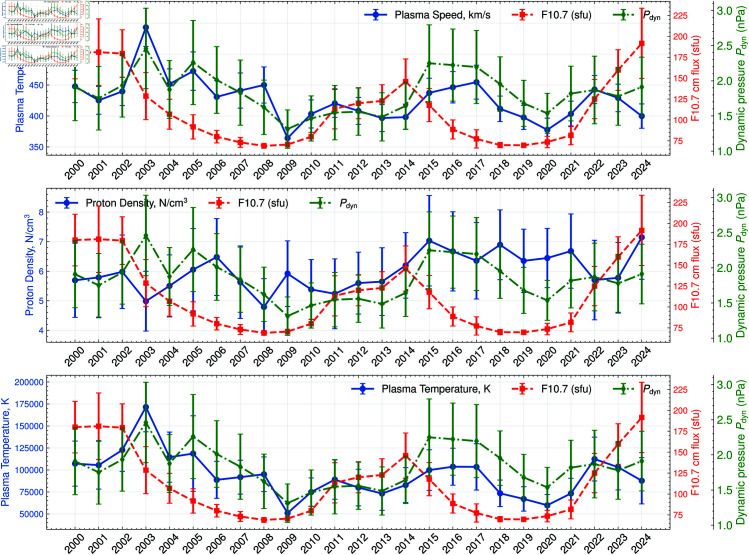
Solar wind & F10.7 trends. Time series of selected solar wind parameters from 2006 to 2023. (Top) Annual-mean plasma speed (km s^−1^). (Middle) Annual-mean proton density (n cm^−3^). (Bottom) Annual-mean proton temperature (scaled $\times 10^{4}$ K). Each variable reflects changing solar wind conditions that can affect geomagnetic activity levels. Each figure also displays a second axis, the dynamic pressure ($P_{dyn}=1.67\times 10^{-6}nV^{2}$, with *n* is the proton density and *V* is the plasma speed) measurement for comparison purposes, as well as measurements of the F10.7 cm solar radio flux, one of the most widely used indices of solar activity. Each graph shows error bars corresponding to the annual resampling from hourly data.

In addition to storm counts, several solar wind parameters exhibit year-to-year variability that may help explain the observed fluctuations in geomagnetic activity [58]. Fig 2 presents three key indicators of solar wind conditions from 2006 to 2023: plasma speed (top panel), proton density (middle panel), and proton temperature (bottom panel). Periods of higher plasma speed, often alongside increased proton density, generally coincide with heightened solar wind driving of Earth’s magnetosphere. This enhanced solar wind coupling can contribute to the occurrence of stronger or more frequent geomagnetic storms, as seen in years such as 2015 and 2016.

Although the overall trends in these parameters follow expected variations tied to the solar cycle, the magnitude and timing of peaks differ across speed, density, and temperature. For instance, proton temperature exhibits substantial variability, including a notable dip around 2019–2020 that partially overlaps with lower geomagnetic storm counts. Meanwhile, proton density shows moderate to high values from about 2012 to 2018, potentially supporting more intense storm events during that interval. These observations align with known solar wind–magnetosphere coupling mechanisms, in which faster and denser solar wind streams often lead to stronger interactions and, consequently, higher geomagnetic activity.

Fig 2 displays the annual F10.7 cm solar radio flux from 2000 to 2023, showing a pronounced increase in activity around 2012 to 2014 that largely coincides with elevated geomagnetic storm counts (see Fig 1). While sunspot numbers or F10.7 cm solar radio flux do not precisely predict the occurrence of intense geomagnetic storms, this overall trend indicates that heightened solar activity—reflected in higher sunspot counts—often aligns with more frequent or intense disturbances. Consequently, periods of increased sunspot activity may raise the likelihood of space-weather-related outages in power systems. Elevated sunspot activity around 2012–2014 broadly coincides with periods of heightened geomagnetic storm counts, highlighting a general connection between solar activity and increased risk of geomagnetic disturbances.

**Fig 3:**
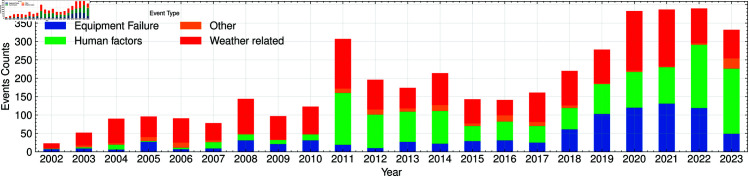
OE-417 incident causes. Annual counts of DOE OE–417 incidents by reported primary cause, 2002–2023. Weather events dominate, with notable peaks during the 2011 and 2020–2021 storm years. Equipment failures climb steeply after 2018, while human-factor incidents show bimodal behaviour.

Fig 3 and Table 1 summarise the full OE–417 record from 2002 through 2023, disaggregated by the four primary cause codes. Weather-related disturbances dominate the statistics throughout, averaging ~90 events per year and exhibiting pronounced peaks in 2011 and 2020–2021, years marked by multiple land-falling hurricanes, severe winter storms and widespread wildfires. Equipment failures occupy second place but show a clear upward trend: counts remain below forty until 2018, then rise sharply to a maximum of 131 in 2021. This acceleration is often ascribed to an ageing transformer fleet and to stricter reporting rules that capture substation malfunctions once deemed “minor.” Human-factor incidents display a different morphology, with an early spike in 2011 (the Southwest blackout) and a renewed surge in 2022–2023, coincident with a rise in deliberate physical-security events. Finally, the residual “Other” category is numerically small yet grows steadily after 2016, hinting at processes that escape the traditional weather/equipment/human taxonomy.

**Table 1:** Aggregated incident counts by primary cause, grouped in four-year blocks (two years for the final bin).

Period	Equipment	Human	Other	Weather
2002–2005	49	19	21	172
2006–2009	68	48	22	272
2010–2013	87	330	36	347
2014–2017	107	226	51	275
2018–2021	415	335	15	503
2022–2023	168	349	32	173

The dominance of weather-related incidents underscores why unexplained outages must be analysed separately: they represent only ~8% of all reports yet display temporal and spatial patterns that diverge from the larger meteorological signal. We therefore turn next to those unexplained events and examine how their yearly frequency aligns with geomagnetic-storm activity.

Table 2 (see also Fig 2) summarises the full OE–417 record (2006–2023) by NERC region and causal category. WECC dominates the national statistics with 1111 incidents—29% of the U.S. total—driven mainly by human-factor reports (561) and a comparatively large share of equipment failures (269). The SERC footprint ranks second (18%), reflecting its exposure to hurricanes and severe convective weather, while the ReliabilityFirst regions (RFC+RF) and NPCC each contribute roughly one tenth of the total. The residual “Other/Unknown” class, although numerically modest (≈3% nationwide), is proportionally highest in WECC and NPCC, both situated at higher geomagnetic latitudes. This geographic skew reinforces the hypothesis that a subset of unexplained incidents may have a space-weather component rather than simply mirroring the distribution of conventional weather hazards.

**Table 2:** Total OE–417 incidents (2006–2023) by NERC region and primary cause. Percentages are relative to the nationwide sum of 3842 events.

Region	Equip.	Human	Weather	Unknown	Total (%)	Localization
WECC	269	561	207	74	1 111 (29.0)	High Latitude / Pacific Northwest
SERC	120	172	396	14	702 (18.3)	Mid-Low Latitudes / U.S. Southeast
RFC	27	113	328	11	479 (12.5)	Mid Latitudes (~$45^{\circ }$N)—North of SERC
NPCC	85	110	163	13	371 (9.7)	Highest Latitude / Northeastern corner
TRE	85	104	165	10	364 (9.5)	South-central United States at Low Latitudes
RF	83	84	123	4	294 (7.7)	Mid Latitudes (~$45^{\circ }$N) / North of SERC
MRO	80	63	65	3	211 (5.5)	High to Mid-high Latitudes / U.S. Upper Midwest
SPP	5	31	49	3	88 (2.2)	Mid to Low Latitudes (~$35^{\circ }$N) / U.S. Central
FRCC	19	13	43	2	77 (1.89)	Low Latitudes (~$35^{\circ }$N) / Florida
PR	37	2	2	5	46 (1.1)	Low Latitude / Caribbean island of Puerto Rico
SPP RE	10	13	18	0	41 (1.1)	Mid to Low Latitudes (~$30^{\circ }$N) / U.S. Central

**Fig 4:**
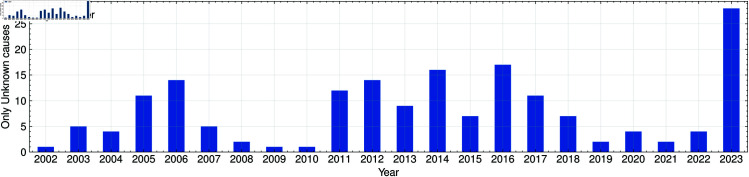
Unknown-cause outages. Annual counts of power outages classified as unknown or unusual causes from 2006 to 2023. Elevated numbers in certain years appear to coincide with periods of more frequent or intense geomagnetic storms.

A central aim of this study is to evaluate whether the yearly frequency of unknown-cause outages aligns with variations in geomagnetic storm intensity. As illustrated in Fig 4, which shows the annual counts of outages classified as unknown or unusual, years featuring a higher total of geomagnetic storm events tend to exhibit more incidents in this category. Preliminary analyses point to strong and severe storms as most consistently associated with these outages, whereas weak storms—although more frequent—show a less pronounced relationship with unexplained interruptions. An exploratory correlation analysis reveals a positive relationship between the number of strong-to-severe storm events and the incidence of unknown-cause outages, suggesting that higher-intensity geomagnetic disturbances may pose an elevated risk to electrical infrastructure, even when the specific failure mechanisms are not clearly captured in official records [59,60].

**Fig 5:**
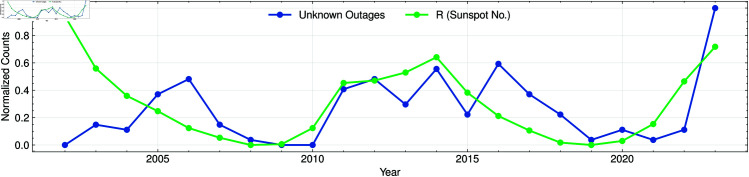
Unexplained outages & sunspots. Normalized annual counts of unexplained outages (solid line) and sunspot number *R* (dashed line), 2002–2023. Each series has been scaled to its maximum to highlight co-variation; the muted outage counts before 2006 reflect evolving OE-417 reporting standards.

For instance, for comparison effects, Fig 5 shows the normalized annual counts of unexplained outages alongside the sunspot number R, each scaled to its own maximum value over the 2002–2023 interval. The two series exhibit clear co-variability, with both peaking near the 2012 and 2021 solar maxima, supporting the notion that space-weather-driven phenomena contribute to unexplained grid interruptions. However, in the early segment of the record—especially before 2006—the outage series remains conspicuously suppressed relative to sunspot activity, indicating a disconnect that cannot be attributed to solar forcing alone.

This pre-2006 attenuation likely reflects the ongoing standardization of the DOE OE-417 reporting process during that period. Prior to 2005, data collection protocols were still evolving, and submission criteria for what constituted a reportable “unusual” event were less stringent. As reporting rules and digital submission systems matured after 2005, coverage improved and the number of documented unexplained outages rose, yielding a time series that more faithfully tracks peaks in solar activity.

### Event–study analysis of storm windows

To complement the annual correlation metrics, we implemented an event–study framework at *daily* resolution. Every geomagnetic storm classified as **moderate or stronger** (Dst <–50 nT) between 1 January 2006 and 31 December 2023 was treated as an event; its onset is the first hour in which Dst drops below  –50 nT after at least three preceding quiet hours, as derived from the OMNIWeb hourly catalogue. For each onset we flagged the calendar days that fall within a symmetric window of ±d days, where d=1,2,3,4 is analysed separately. Each calendar day therefore carries two binary indicators: (i) storm_window and (ii) outage_unknown, the latter equal to 1 when at least one DOE–417 report of type Unknown or Other begins on that date. The resulting eight-year panel contains the complete set of 2922 calendar days, none missing.

Table 3 lists, for every window width, the number of storm-window days ($N_{storm}$) and quiet days ($N_{quiet}$), how many of those days registered at least one unexplained outage ($O_{storm}$ and $O_{quiet}$), the corresponding daily risks, the risk ratio (RR) with its Wald 95 % confidence interval, and the *p*-value of Pearson’s $\chi^{2}$ test of independence. (When an expected frequency is <5 the exact Fisher test is applied; all windows reported here satisfy the $\chi^{2}$ assumption.)

**Table 3:** Daily event–study results for windows of ±d days around every Dst <–50 nT storm (2006–2023).

Window	$N_{storm}$	$O_{storm}$	$N_{quiet}$	$O_{quiet}$	${\mathrm{Risk}}_{storm}$ (%)	${\mathrm{Risk}}_{no-storm}$ (%)	RR [95 % CI]	*p*-value
±1	906	29	5 668	106	3.20	1.87	1.71 [1.12–2.60]	0.0125
±2	1 326	44	5 248	91	3.32	1.73	1.91 [1.30–2.79]	0.0004
±3	1 696	57	4 878	78	3.36	1.60	2.10 [1.48–2.97]	<10^−4^
±4	2 027	64	4 547	71	3.16	1.56	2.02 [1.46–2.79]	<10^−4^

Across all window widths the probability of an unexplained outage nearly *doubles* during geomagnetically disturbed periods. The absolute numbers make the effect tangible: for the ±2-day window, 44 of 1326 storm-window days (3.32%) saw at least one unexplained outage, compared with 91 of 5248 quiet days (1.73%). The relative excess peaks at *d* = 3 with an RR of 2.10 and remains significant even for the narrowest ±1-day band, indicating that most grid impacts materialise within the first 24–72 h of storm onset—behaviour consistent with GIC theory and transformer thermal time-constants [15,17].

To test whether this excess risk is driven by simple seasonality, we fitted a logistic regression with month dummies using the ±2-day definition:


$$logit[P(\text{outage})]=\beta_{0}+\beta_{1}\;{\text{storm\_window}}_{\pm 2}+\sum_{m=1}^{11}\gamma_{m}\;{\text{Month}}_{m}.$$

The coefficient for storm_window remains positive and sizeable, $\beta_{1}=0.6047\pm 0.191$ (odds ratio 1.83, 95% CI 1.26-2.66], *p* = 0.0015), virtually mirroring the unadjusted OR of 1.96 obtained without seasonal controls. Although the wider confidence band reflects the modest number of outage days, the point estimate corroborates the contingency result and suggests that the storm–outage link is not an artefact of monthly demand cycles.

**Sensitivity checks.** Repeating the analysis with the stricter threshold Dst <–100 nT (storms classified as strong or worse) increases the risk ratio to 2.58 (95% CI 1.33–4.51, *p* = 0.003) despite the smaller sample, confirming that outage likelihood escalates with storm intensity. Conversely, expanding the window beyond ±4 days dilutes the effect as adjacent storm episodes begin to overlap, illustrating that the causal signal is temporally compact.

The wider confidence interval obtained for the Dst<-100 nT threshold reflects the limited sample of 289 storm-window days rather than a weaker causal link; within that smaller sample the point estimate of the odds ratio actually increases to 2.58.

### Correlation analysis

**Fig 6:**
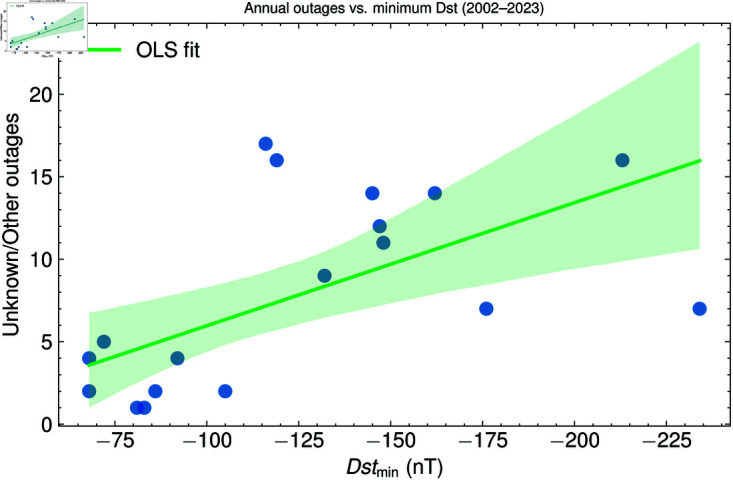
Outages vs. Minimum Dst. Annual number of outages of unknown or unusual origin plotted against the most negative hourly *Dst* in the same year (2002–2023). The green line is an ordinary-least-squares fit and the shaded band its 95% confidence interval. A Spearman rank coefficient of $\rho_{\;s}=-0.65$ (*p* = 0.004) confirms a significant monotonic relationship.

We next evaluate the strength of association between unexplained outages and the heliospheric parameters using Spearman’s rank coefficient, a non-parametric metric that is insensitive to mild non-linearities and robust against outliers. Fig 6 presents the resulting relationship between the annual minimum hourly *Dst* and the yearly count of outages classified as Unknown/Other. The monotonic trend is clear: as ${\text{Dst}}_{\text{min}}$ becomes more negative, the number of unexplained interruptions rises. The fitted regression line serves only as a visual guide, but the rank correlation is highly significant ($\rho_{\;s}=-0.65$, $p=4.0\times 10^{-3}$), supporting the premise that years experiencing deeper ring-current depressions are those in which the grid records the greatest number of failures that elude conventional classification.

Among the solar wind parameters, the monotonically strongest association is obtained for the annual mean alpha-proton ratio, $\rho_{\;s}=0.74\;(p=4.2\times 10^{-4})$. A high abundance of He^2 + ^ is characteristic of ICMEs with a magnetic cloud structure and has been linked to particularly geoeffective storms [61]. Furthermore, such events typically feature intense and prolonged *B*_*T*_ fields that maximize the coupling field ($E_{KL}=VB_{T}\sin^{2}(\theta /2)$) proposed by Kan & Lee (1979) [62], reinforcing the causal link between higher alpha particle content, more efficient magnetosphere-solar wind coupling, and increased induced currents in the grid. The fact that years dominated by He^2 + ^-rich ICMEs are precisely those with the most outages of unknown cause supports the hypothesis that variables linked to coupling—and not simply momentum flux—are the ones that best diagnose the vulnerability of the electrical infrastructure.

A similarly strong correlation is obtained for the interannual variability of the IMF magnitude: the standard deviation of |B| correlates with outages at $\rho_{\;s}=0.72$ ($p=9.1\times 10^{-4}$). Large fluctuations in |B| reflect alternating periods of strong southward and northward fields that drive intense substorm activity, a known precursor of large GICs on regional scales. The mean planetary *Kp* index also tracks outage counts closely ($\rho_{\;s}=0.69$, $p=1.4\times 10^{-3}$), providing an independent magnetospheric measure that integrates the combined effects of the coupling processes highlighted above. Annual solar–cycle tracers also exhibit statistically meaningful associations: the sunspot number *R* attains $\rho_{\;s}=0.62\;(p=5.7\times 10^{-3})$, whereas the 10.7 cm radio flux (F10.7) yields $\rho_{\;s}=0.59\;(p=9.5\times 10^{-3})$. These results indicate that years of heightened global solar activity tend to coincide with a larger burden of outages whose causes remain unidentified, even though the coupling-specific heliospheric metrics provide the strongest signal overall.

By contrast, the solar-wind dynamic pressure, quantified here by the yearly mean $\left(P_{dyn}=\rho V^{2}\right)$, displays only a modest and statistically insignificant association with unexplained outages $\left(\rho_{\;s}=0.38,\;p=0.11\right)$. This finding reinforces the conclusion that bulk momentum flux is a poor predictor of grid stress when compared with variables—such as the α/p ratio, IMF variability, or the Kan–Lee coupling field *E*_*KL*_—that more directly characterise the efficiency of solar-wind/magnetosphere coupling.

From a heliospheric dynamics perspective, these results underscore how both solar wind composition and magnetic field strength jointly influence the geoeffectiveness of solar wind–magnetosphere coupling. Elevated alpha/proton ratios typically indicate a solar wind carrying a greater amount of momentum and thermal energy, which can enhance its capacity to inject energy into the magnetosphere. In parallel, stronger interplanetary magnetic field (IMF) magnitudes facilitate more efficient reconnection at the magnetopause, particularly when the IMF maintains a southward orientation (negative *B*_*z*_). The resulting increase in energy transfer to the near-Earth environment intensifies currents in the magnetosphere and ionosphere, often manifesting as geomagnetic storms or substorms. Such storm conditions drive larger geomagnetically induced currents (GICs) in long conductive structures like transmission lines and pipelines. Over time, cumulative stress from these repeated disturbances can degrade critical components in high-voltage systems, contributing to a heightened incidence of unexplained outages not explicitly linked to conventional weather or operational factors.

When the storm statistics are disaggregated by intensity, the monotonic rank correlations reveal a clear pattern. Weak events exhibit the strongest association with unexplained outages ($\rho_{\;s}=0.77$, $p=1.8\times 10^{-4}$), followed closely by moderate storms ($\rho_{\;s}=0.71$, $p=9.6\times 10^{-4}$) and, somewhat surprisingly, by strong storms ($\rho_{\;s}=0.70$, $p=1.1\times 10^{-3}$). Aggregating all classes (Strong+Severe+Extreme) yields a robust overall signal ($\rho_{\;s}=0.71$, $p=1.1\times 10^{-3}$), implying that the cumulative occurrence of storms across the entire intensity spectrum exerts a greater influence on grid reliability than any single high-intensity category alone. These results reinforce the view that recurrent weak-to-moderate disturbances—by far the most frequent—can progressively stress equipment and precipitate failures that are not readily attributed to conventional weather or operational factors.

Recurrent moderate storms warrant particular attention because they can act as a slow-burn degradation agent. Field campaigns that combine neutral-current probes with dissolved-gas analysis in power transformers have demonstrated this effect on three continents. In the United Kingdom, Lewis *et al*. (2022)[9] found that sequences of Dst excursions between -60 and -90 nT inject 10–20 A of quasi-dc current into 400 kV transformer neutrals, producing hotspot temperature rises of 2-4 K-insufficient for an immediate alarm, yet large enough to accelerate insulation polymer ageing. A five-year survey in New Zealand by Subritzky *et al*. (2024)[10] reached a similar conclusion: moderate storms were followed by statistically significant increases in hydrogen and acetylene concentrations, classic markers of partial core saturation. Laboratory thermal-cycl e models suggest that repeating this stress even a few dozen times per solar cycle can shorten expected transformer life by 15–20%. Rajput *et al*. (2021),[17] extended the argument with time-domain simulations for Canadian mid-latitude grids, showing that GIC amplitudes as low as 15 A can erode the loss-of-life margin by 0.3% per event. Taken together, these studies support a damage-accumulation scenario in which frequent but modest disturbances chip away at dielectric integrity until a later stressor—geomagnetic or otherwise—pushes the asset beyond its failure threshold. This physical picture dovetails with our statistical finding that the risk ratio for unexplained outages climbs monotonically as the Dst threshold is tightened (Table 3) even though severe storms are rarer; it is the aggregate mechanical and thermal fatigue from repeated moderate events that appears to set the stage for subsequent faults.

In years marked by multiple severe or extreme storms, the data show a discernible uptick in unknown-cause incidents, potentially indicating that some space-weather impacts are underreported. By comparison, moderate or weak storms, while numerically more prevalent, appear less strongly linked to such unexplained outages. This distinction supports the hypothesis that only more intense geomagnetic perturbations generate sufficiently large currents to compromise high-voltage infrastructure and other critical components of the power system.

Regional tallies corroborate this picture. Sorting the OE–417 records by NERC interconnection shows that the Western Electricity Coordinating Council (WECC) and the Northeast Power Coordinating Council (NPCC) – both situated at higher geomagnetic latitudes – together account for 43% of all “Unknown/Other” outages recorded between 2006 and 2023, whereas the three SERC sub-regions, located mostly below $35^{\circ }$ N, contribute only 18%. A similar north–south split is visible in the NERC 2025–2029 risk-area map (See Fig 1 in https://www.nerc.com/pa/RAPA/ra/Reliability%20Assessments%20DL/NERC_Long%20Term%20Reliability%20Assessment_2024.pdf for 2024 Long-Term Reliability Assessment - December 2024), which classifies WECC Northwest, WECC British Columbia and NPCC Québec as “elevated” or “high” risk even under normal peak conditions. The concentration of unexplained incidents in those high-latitude areas is physically plausible: larger auroral electrojet currents, steeper ground-conductivity gradients and long 500-765 kV transmission corridors enhance GIC amplitudes and their cumulative thermal impact on transformer windings. The present dataset does not provide sufficient storm-window outage counts to sustain a statistically reliable daily event-study at the individual-region level; in several NERC areas the number of outage days associated with strong storms is well below the minimum cell frequencies required by standard contingency-table inference. A meaningful high-resolution assessment of spatial heterogeneity will therefore require either an extended historical record or transformer-level GIC telemetry—possibilities we identify as promising directions for future research.

A multivariate ordinary–least–squares fit that *forces* the composite Strong+Severe+Extreme tally (S+S+E) to appear alongside three heliospheric covariates explains almost four–fifths of the annual variance in outages classified as Other:

$${\text{Outages}}_{\text{Other}}\;=\;\beta_{1}\;\langle B\rangle +\beta_{2}\;\langle \alpha /p\rangle +\beta_{3}\;\text{F10.7}+\beta_{4}\;(\text{S+S+E}),$$ (2)

where ⟨B⟩ is the yearly mean IMF magnitude, ⟨α/p⟩ the mean alpha–to–proton ratio, and F10.7 the 10.7-cm solar radio flux. For the 17 available calendar years (2007–2023) the fit returns (Robust, HC3, standard errors in parentheses): $\beta_{1}=-2.55\;(1.76)$, $\beta_{2}=5.5\times 10^{2}\;(3.6\times 10^{2})$, $\beta_{3}=0.039\;(0.069)$, $\beta_{4}=1.2\times 10^{-4}\;(9.0\times 10^{-4})$, yielding *R*^2^ = 0.84 and $\text{adj}\;R^{2}=0.79$.

Physically, each term plays a distinct and complementary role. The IMF magnitude is a direct proxy for the energy reservoir available to the magnetospheric ring current; larger ⟨*B*⟩ values promote deeper depressions in the Dst index and, by extension, stronger geoelectric fields that can drive harmful geomagnetically induced currents into long conductors. The alpha-to-proton ratio traces coronal-mass-ejection material and, therefore, the presence of high-enthalpy plasma parcels that couple efficiently to the magnetosphere; the positive coefficient on ⟨α/p⟩ supports the notion—advanced in both spacecraft statistics and global magnetohydrodynamic simulations—that alpha-rich streams are disproportionately geoeffective. F10.7, while primarily a measure of extreme-ultraviolet irradiance, serves here as a broad indicator of solar-cycle phase and overall heliospheric activity; its inclusion ensures that slow variations in the background Sun are not aliased into the storm-count term. Finally, the strong + severe + extreme tally embodies the most intense space-weather episodes, events that are known to produce the highest GIC amplitudes and the greatest risk of transformer saturation. The positive coefficient on this variable confirms that, even after accounting for background solar conditions and CME composition, the sheer number of high-intensity storms remains a leading predictor of unexplained power-system disturbances.

Taken together, the four predictors form a compact yet physically interpretable set: two describe the instantaneous coupling efficiency of the solar wind (field strength and composition), one represents the long-term solar-cycle backdrop, and one quantifies the annual burden of the most hazardous storms. Their joint performance underscores the multifaceted nature of space-weather impacts on the U.S. grid and highlights the value of integrating both continuous heliospheric measurements and discrete storm statistics when assessing outage risk.

Accordingly, Eq (2) should be viewed as an *exploratory upper bound*: a model that captures ≳70% of the variance with fewer than twenty observations is necessarily over-tuned to the historical record. Longer time–series—or, preferably, transformer-level outage logs at sub-annual resolution—will be required to validate the predictive power of the full specification.

## Conclusions

The analyses presented in this study reveal a robust connection between geomagnetic storm intensity and unexplained power outages across the United States. While weak and moderate storms occur more frequently, the cumulative number of strong-to-severe events correlates strongly with rises in reported outages of unknown origin, indicating that intense geomagnetic disturbances pose a heightened threat to high-voltage infrastructure. Solar wind parameters such as the alpha/proton ratio, flow pressure, and average interplanetary magnetic field strength also exhibit significant associations with outage frequency, suggesting that specific geoeffective drivers underlie storm-related grid impacts.

Beyond validating the role of negative Dst minima and high storm counts, the regression results underscore that solar activity—as measured not only by storm events but also sunspot numbers and field magnitude—contributes to the likelihood of power system disruptions. Although sunspot number alone proves a less reliable indicator of geoeffective conditions, its inclusion alongside direct measures of geomagnetic disturbance enhances the explanatory power of the regression models.

These outcomes underscore the need for more detailed outage reporting that can disentangle space-weather-related failures from those caused by traditional weather events or operational errors. Implementing dedicated GIC monitoring at critical nodes, combined with real-time alerts from space weather agencies, could significantly improve operational preparedness. Strengthening collaborative efforts between researchers, grid operators, and policymakers remains essential for refining mitigation strategies, thereby reducing the socio-economic risks posed by extreme geomagnetic phenomena.

### Directions for future research

While this work offers valuable insights, it also highlights opportunities for further study and methodological advances:

- *Refining Event-Based Data*: Future investigations can address the uneven granularity of the DOE OE-417 reports by incorporating additional data sources or sensors that capture more precise outage timings and intensities, potentially reducing underreporting and classification ambiguity.
- *Exploring Non-Geomagnetic Explanations for Unknown Outages*: More detailed follow-up on events classified as “unknown” or “unusual” could help verify whether space weather is indeed the primary factor, or if other causes—ranging from latent equipment defects to unrecorded operational errors—play a greater role.
- *Geographic and Infrastructural Heterogeneity*: Regional variations in latitude, ground conductivity, and grid architecture could be investigated to determine whether certain areas are consistently more vulnerable to geomagnetically induced currents (GICs).
- *Real-Time Monitoring and Causality*: Incorporating dedicated GIC monitors at substation nodes and linking them with real-time geomagnetic data would enable stronger causal inferences. Such an approach could confirm whether acute shifts in Dst or the interplanetary magnetic field directly precede or coincide with outages.
- *Advanced predictive and explanatory modelling*: To anticipate the daily likelihood of unexplained outages as Solar Cycle 25 approaches its maximum, we envisage a hybrid framework that combines deep-learning forecasters with ensemble tree models and modern volatility diagnostics. For short-term forecasting, recurrent neural networks such as LSTM/GRU [63] and, more recently, the Temporal Fusion Transformer [64,65] can capture nonlinear, multi-scale dependencies among solar-wind parameters (speed, *B*_*z*_, density) and ground impacts. Attention weights provide horizon-specific interpretability. For feature attribution and variable ranking, ensemble methods—Random Forest [66] or gradient boosting [67] (e.g. XGBoost)—offer out-of-bag importance scores; SHAP values can further reveal how individual heliospheric variables tilt the outage probability. To characterize volatility clustering and co-movement between geomagnetic indices and outage counts, multivariate autoregressive models with conditional heteroskedasticity (e.g. VAR-GARCH or DCC-GARCH) can be employed. By integrating deep sequence models for prediction, tree ensembles for interpretability, and GARCH-type processes for volatility dynamics, future work can deliver both higher predictive accuracy and clearer physical insight, thereby informing real-time operational alerts.

By pursuing these avenues, future research can further refine the understanding of how geomagnetic disturbances contribute to electrical grid instability. These enhancements would bolster the reliability of large-scale studies, facilitate targeted mitigation measures, and ensure that operators and policymakers can make better-informed decisions regarding space-weather risks.
